# Supplementary material for: Forest Fire Clustering for single-cell sequencing combines iterative label propagation with parallelized Monte Carlo simulations
Source: Nat Commun. 2022 Jun 20;13:3538. doi: 10.1038/s41467-022-31107-8 (PMC9209427; doi:10.1038/s41467-022-31107-8)
Supplement: Supplementary file 1 — Supplementary Information (PDF file) [file 41467_2022_31107_MOESM1_ESM.pdf]

Supplementary Text and Figures for "Forest Fire Clustering for Single-cell Sequencing Combines Iterative Label Propagation with Parallelized Monte Carlo Simulations"

List of Figures

Supplementary Figure 1 All Datasets Used for Analysis . . . 2
Supplementary Figure 2 CPU Used for Benchmarking . . . 3
Supplementary Figure 3 Illustration of Online Learning . . . 4
Supplementary Figure 4 Online Learning on Simulated Gaussian Mixtures . . . 5
Supplementary Figure 5 Abbreviations of Clustering Algorithms . . . 6
Supplementary Figure 6 Comparison with Existing Methods on Synthetic Data . . . 7
Supplementary Figure 7 PBMC Classical Monocyte Detection Threshold . . . 8
Supplementary Figure 8 Online Learning on Ablated PBMC Data . . . 9
Supplementary Figure 9 Online Learning Classical Monocyte Detection Threshold . . . 10
Supplementary Figure 10 Sankey Diagram of Forest Fire Clusters with Internal Validation . . . 11
Supplementary Figure 11 Clustering Quality on Skin SHARE-seq . . . 12
Supplementary Figure 12 Clustering Quality on Human Cortex . . . 13
Supplementary Figure 13 Cell Path Diffusion Pseudo-time Analysis . . . 14
Supplementary Figure 14 1.3 Million Mouse Brain Cell Runtime and Peak Memory Usage Benchmarks 15
Supplementary Figure 15 Differentially Expressed Genes in Early vs. Late MCA Cells . . . 16
Supplementary Figure 16 Function of Top Differentially Expressed Genes in Early MCA Cells . 17
Supplementary Figure 17 Monte Carlo Runtime and Parallelization Analysis . . . 18
Supplementary Figure 18 Clustering Quality on PBMC for Different K in KNN Graphs . . . 19
Supplementary Figure 19 Clustering Quality on PBMC for Various Dimensionality Reduction Methods . . . 20
Supplementary Figure 20 Posterior Exclusion Probability for Doublet Detection and Quality Control 21
Supplementary Figure 21 Harmony Batch Correction on PBMC with Cell Surface Labels . . . 22
Supplementary Figure 22 Flowchart of the Preprocessing Steps . . . 23
Supplementary Figure 23 Forest Fire Clustering Hyperparameter Configurations on Clustering . 24
Supplementary Figure 24 Forest Fire Clustering Hyperparameter Configurations for Benchmarking 25

Source	Assay Type	Tissue	Labels	# of Cells
Paul et al., 2015	scRNA-seq	Mouse Hematopoietic Stem Cells	Known Marker Genes	2,700
10x Genomics	CITE-seq (scRNA + Antibody-seq)	PBMC	Cell Surface Antibody Capture	10,000
10x Genomics	Single-cell Multiome (scATAC + scRNA-seq)	PBMC	Known Marker Genes	10,000
Hodge et al., 2019	SMART-seq (snRNA-seq)	Human Cortex	Known Marker Genes	15,928
Moon et al., 2019	scRNA-seq	Human Embryonic Stem Cells	Real Developmental Days	16,825
Ma, et al., 2020	SHARE-seq (single-cell multiomics scATAC + scRNA-seq)	Skin	Known Marker Genes	84,426
Han et al., 2018	Microwell-seq (scRNA-seq)	Mouse Cell Atlas	Tissue Annotations	400,000
10x Genomics	scRNA-seq	Mouse Brain Cells	Known Marker Genes	1,300,000

Supplementary Figure 1. All Datasets Used for Analysis: All of the experimental single-cell sequencing datasets used to benchmark the accuracy, runtime, and peak memory usage of Forest Fire Clustering.

```
Architecture:          x86_64
CPU op-mode(s):        32-bit, 64-bit
Byte Order:            Little Endian
CPU(s):                36
On-line CPU(s) list:   0-35
Thread(s) per core:    1
Core(s) per socket:    18
Socket(s):             2
NUMA node(s):          4
Vendor ID:             GenuineIntel
CPU family:            6
Model:                 85
Model name:            Intel(R) Xeon(R) Gold 6240 CPU @ 2.60GHz
Stepping:              7
CPU MHz:               999.914
CPU max MHz:           3900.0000
CPU min MHz:           1000.0000
BogoMIPS:              5200.00
Virtualization:        VT-x
L1d cache:             32K
L1i cache:             32K
L2 cache:              1024K
L3 cache:              25344K
NUMA node0 CPU(s):     0,4,8,12,16,20,24,28,32
NUMA node1 CPU(s):     1,5,9,13,17,21,25,29,33
NUMA node2 CPU(s):     2,6,10,14,18,22,26,30,34
NUMA node3 CPU(s):     3,7,11,15,19,23,27,31,35
```

Supplementary Figure 2. CPU Used for Benchmarking: CPU used to benchmark the runtime and memory usage of Forest Fire Clustering versus other clustering algorithms.

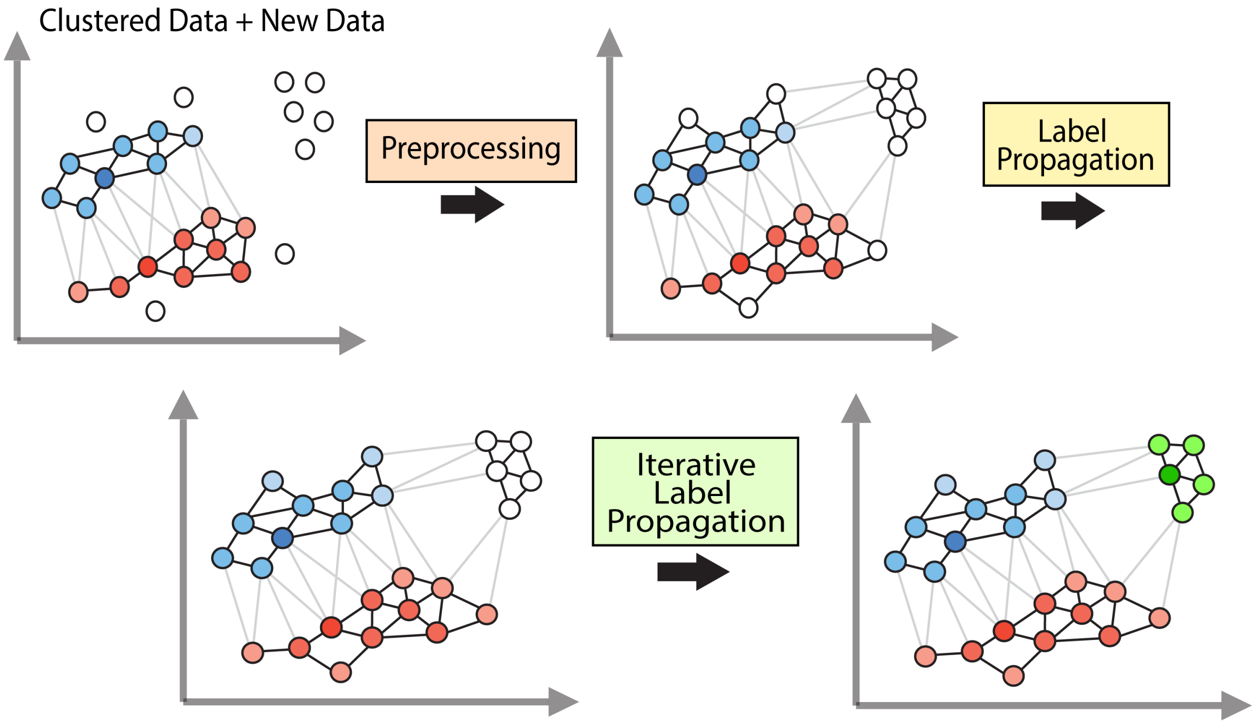

Supplementary Figure 3. Illustration of Online Learning: Given new data, we iteratively check whether label influences from existing clusters can cross the threshold of the new vertex. If multiple label influences from different clusters exceed the threshold, then the new data point takes on the label with the highest average influence. If none of the average label influences can cross the threshold, then the data point becomes the seed vertex for a new cluster. Hence, our framework can discover new clusters in newly arrived data without re-clustering, which reduces computational cost when analyzing large datasets.

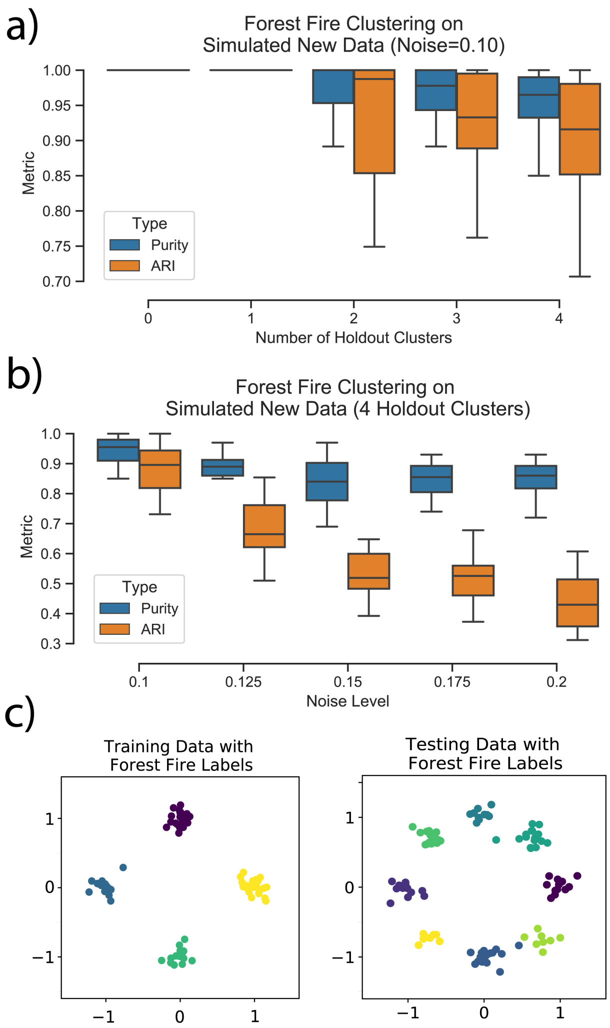

Supplementary Figure 4. Online Learning on Simulated Gaussian Mixtures: a) Performance of Forest Fire Clustering as the number of holdout clusters increased with $\sigma = 0.1$. b) Performance of Forest Fire Clustering as the noise increased with four holdout clusters in the testing data. c) Example of training data containing Gaussian mixtures $\sigma = 0.1$ with four clusters centered on the unit circle with $n = 500$ points; testing data containing Gaussian mixtures $\sigma = 0.1$ with eight clusters centered around the unit circle $n = 500$ points, four of which were not presented during training. Box plot visualizes data based on the minimum as lower whisker, first quartile or 25 percentile as lower bound of box, median as center, third quartile or 75 percentile as higher bound of box, and maximum as higher whisker.

Abbreviation	Clustering Algorithm	Citation
Forest Fire	Forest Fire Clustering	-
K-Means	K-Means Clustering	Hartigan, John A., and Manchek A. Wong. "Algorithm AS 136: A k-means clustering algorithm." Journal of the royal statistical society. series c (applied statistics) 28.1 (1979): 100-108.
Mean Shift	Mean Shift Clustering	Cheng, Yizong. "Mean shift, mode seeking, and clustering." IEEE transactions on pattern analysis and machine intelligence 17.8 (1995): 790-799.
Affin. Prop.	Affinity Propagation Clustering	Dueck, Delbert. Affinity propagation: clustering data by passing messages. Toronto: University of Toronto, 2009.
Birch	Birch Clustering	Zhang, Tian, Raghu Ramakrishnan, and Miron Livny. "BIRCH: an efficient data clustering method for very large databases." ACM sigmod record 25.2 (1996): 103-114.
GMM	Gaussian Mixture Model Clustering	McLachlan, Geoffrey J., and Kaye E. Basford. Mixture models: Inference and applications to clustering. Vol. 38. New York: M. Dekker, 1988.
Ward	Ward Clustering	Murtagh, Fionn, and Pierre Legendre. "Ward's hierarchical agglomerative clustering method: which algorithms implement Ward's criterion?" Journal of classification 31.3 (2014): 274-295.
Agglo. Clust.	Agglomerative (Hierarchical) Clustering	Johnson, Stephen C. "Hierarchical clustering schemes." Psychometrika 32.3 (1967): 241-254.
DBSCAN	Density-Based Spatial Clustering of Applications with Noise	Ester, Martin, et al. "A density-based algorithm for discovering clusters in large spatial databases with noise." KDD. Vol. 96. No. 34. 1996.
OPTICS	Ordering Points To Identify Cluster Structure	Ankerst, Mihael, et al. "OPTICS: Ordering points to identify the clustering structure." ACM Sigmod record 28.2 (1999): 49-60.
Spectr. Clust.	Spectral Clustering	Ng, Andrew Y., Michael I. Jordan, and Yair Weiss. "On spectral clustering: Analysis and an algorithm." Advances in neural information processing systems. 2002.
Louvain	Louvain Community Detection	De Meo, Pasquale, et al. "Generalized louvain method for community detection in large networks." 2011 11th international conference on intelligent systems design and applications. IEEE, 2011.

Supplementary Figure 5. Abbreviations of Clustering Algorithms: Abbreviation, full name, and citation for clustering algorithms used to benchmark in Figure 3 of the main text.

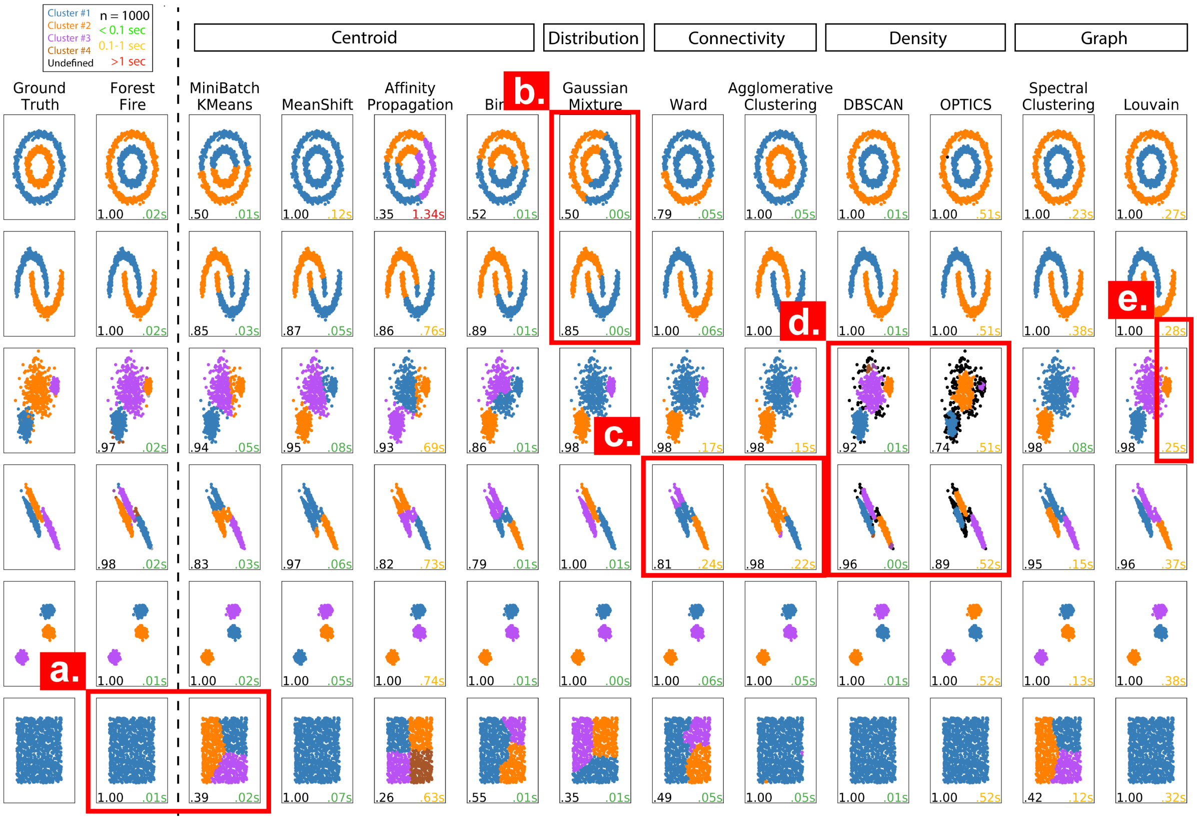

Supplementary Figure 6. Comparison with Existing Methods on Synthetic Data: a) Compared to centroid-based methods, Forest Fire Clustering is not biased towards discovering K clusters from the data. b) Distribution-based methods cannot deal with data that is not well-distributed, since the labels cut across clusters for ring-shaped and half-moon-shaped data. c) Connectivity-based clustering cannot clearly define cluster edges when the intra-cluster distance is larger than inter-cluster distance. d) Density-based clustering outputs singleton points in black. e) Graph-based methods are slow compared other methods.

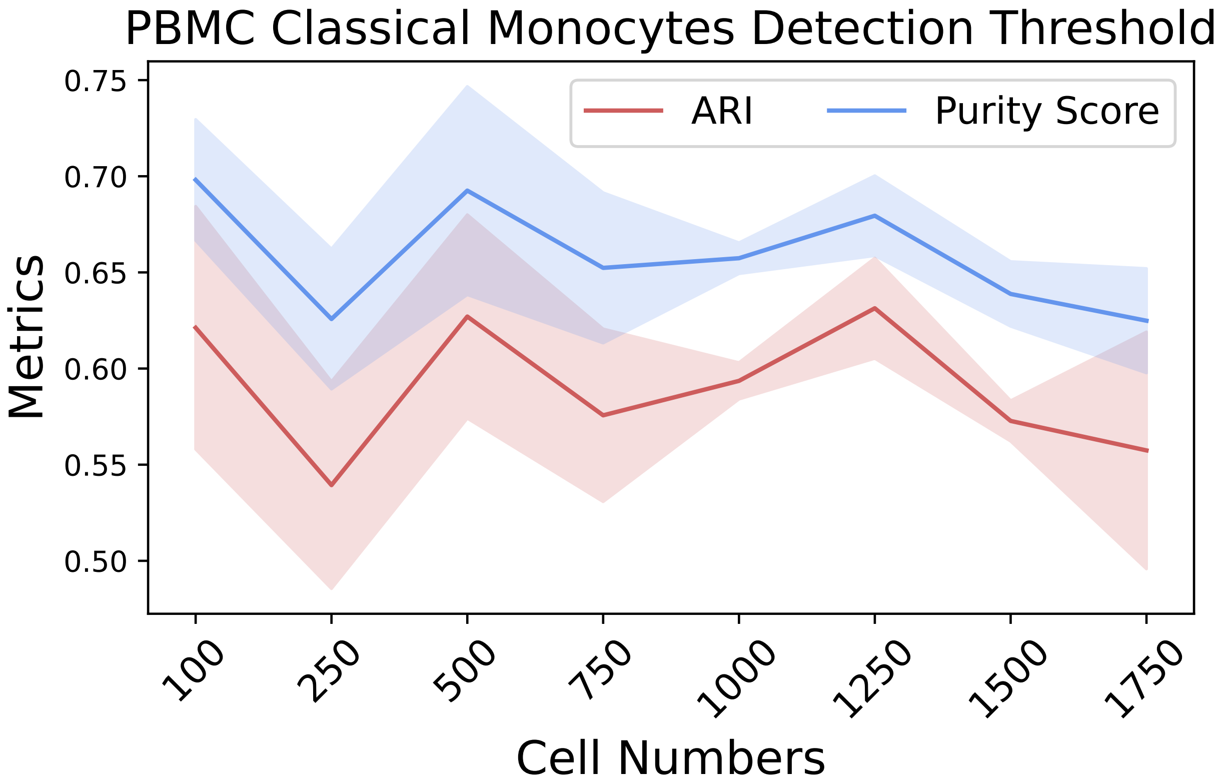

Supplementary Figure 7. PBMC Classical Monocyte Detection Threshold: When gradually removing classical monocytes from the PBMC data (with cell surface labels), Forest Fire Clustering can still robustly discover a cluster even if the cell population is small. Data are presented as mean values +/- 2SD.

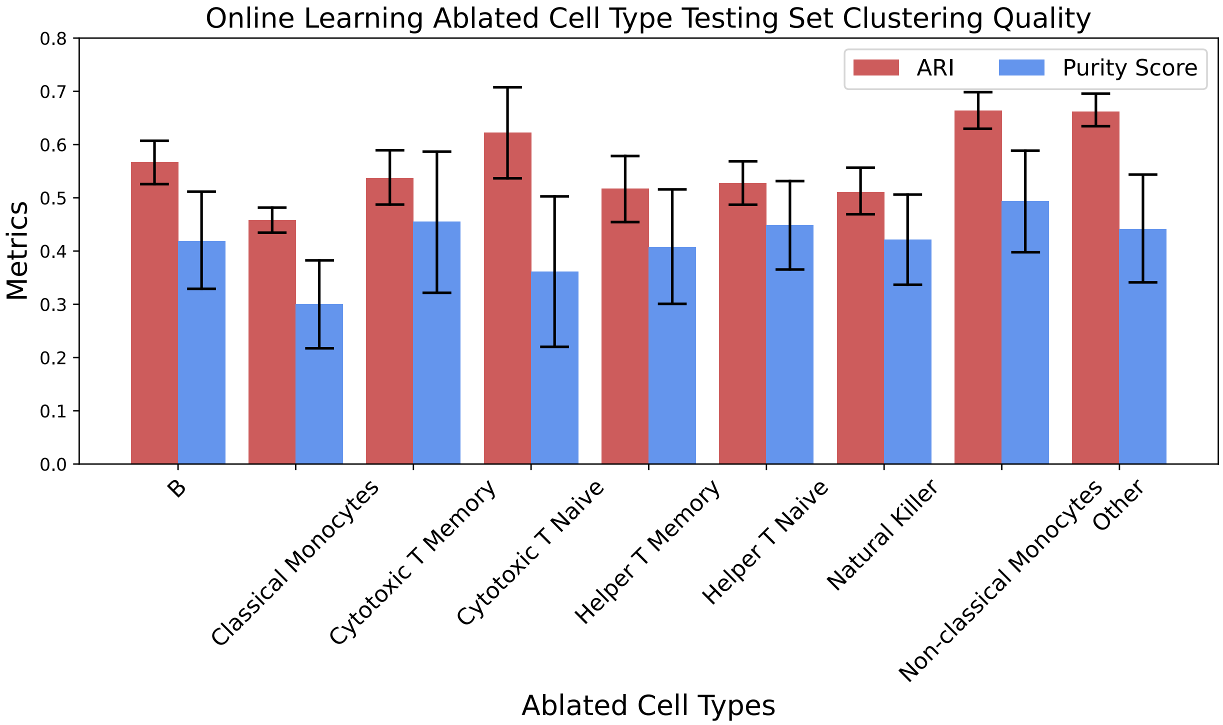

Supplementary Figure 8. Online Learning on Ablated PBMC Data: Each surface protein labeled cell type was ablated from the training dataset, and the rest of the data was split into training and testing with a 9:1 ratio. Then, the ablated cell type was added to the testing dataset. The quality metrics indicate that we could discover new cell types in the testing dataset. Further, to correct for batch effects between independently preprocessed training and testing data, we utilized mutual nearest neighbor correction (MNNs) [1]. MNNs uses the training dataset as a reference dataset and aligns new incoming data onto the reference dataset, which makes it suitable for batch correction in an online-learning setting. Data are presented as mean values +/- 2SD.

In the online learning PBMC experiments, the training and testing datasets were split with a 9:1 ratio after a cell type was ablated, and the ablated cell type was added back to the testing data to evaluate whether Forest Fire Clustering can discover this new unseen cell type. We clustered the training data ($\sigma = 0.1, c = 40$) and used mutual nearest neighbors batch correction ($K = 200$) to align the preprocessed testing count matrix to the training count matrix.

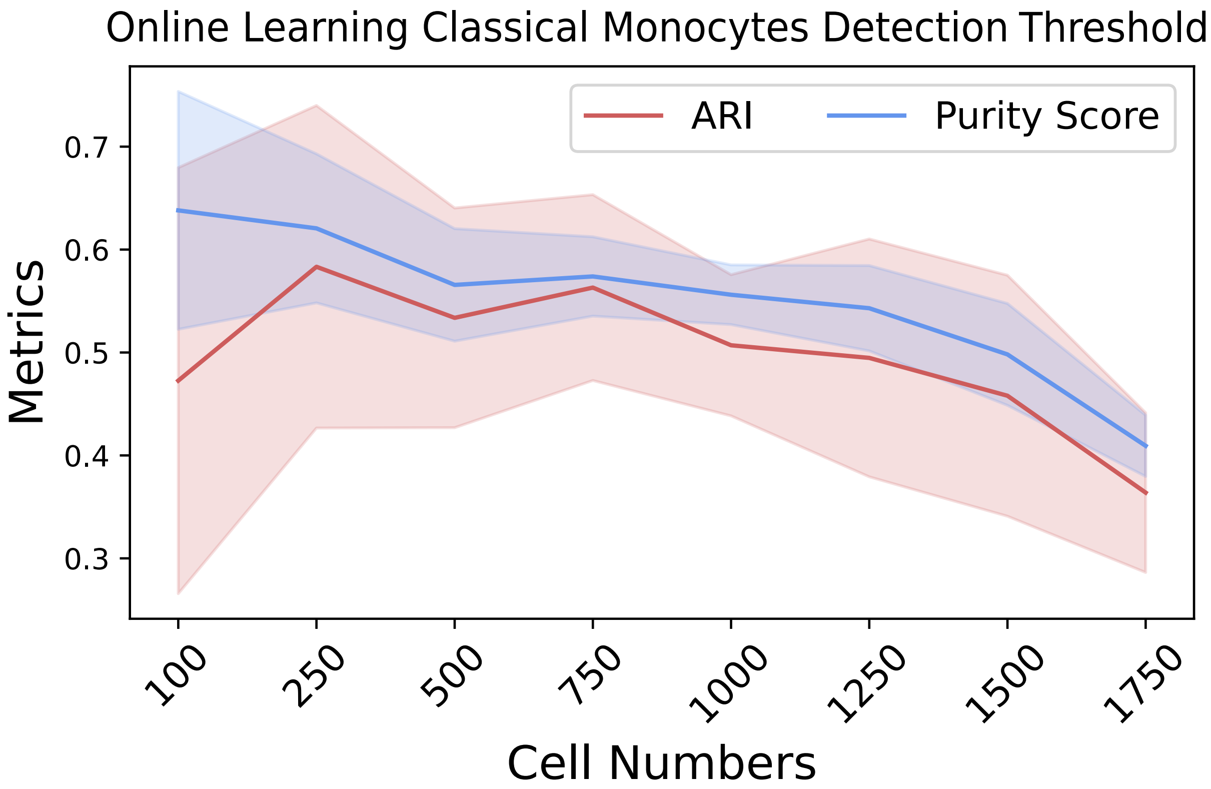

Supplementary Figure 9. Online Learning Classical Monocyte Detection Threshold: In the PBMC data (with cell surface labels), we removed the classical monocytes from the training data and gradually added classical monocytes to the testing data. The results suggest that Forest Fire Clustering can robustly discover small cell populations in online learning. Data are presented as mean values +/- 2SD.

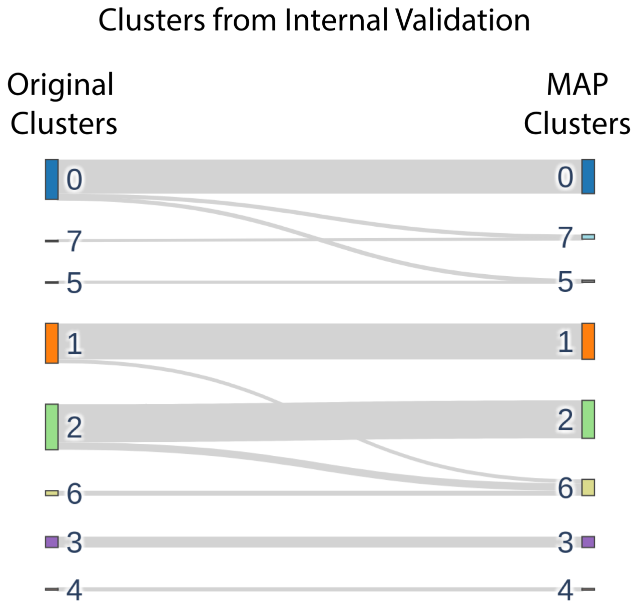

Supplementary Figure 10. Sankey Diagram of Forest Fire Clusters with Internal Validation: After clustering and internal validation on the PBMC cell-surface-protein label dataset, the cluster label with the largest posterior probability was selected as the maximum a posteriori (MAP) label. The results show that 90% of the MAP clusters correspond to the original clusters, indicating that the original set of Forest Fire clustering results is accurate in the context of internal validation.

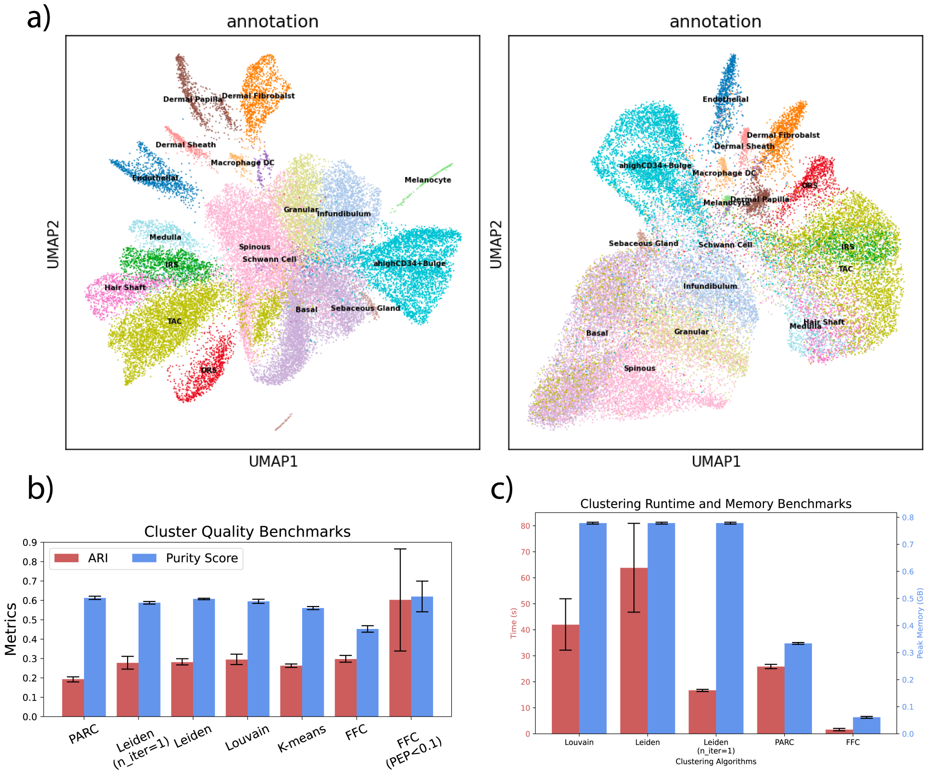

Supplementary Figure 11. Forest Fire Clustering Quality on Skin SHARE-seq: a) Gene expression and chromatin accessibility with cell type annotations. Cell types annotated using marker genes were visualized on UMAP embeddings of gene expression (left) and chromatin accessibility (right). b) Forest Fire Clustering obtained similar quality clusters compared to existing methods, but Forest Fire significantly improved cluster quality by focusing on cells with high confidence labels ($n = 20$ different seeds). c) Runtime and memory usage benchmarks indicate that Forest Fire Clustering is faster and more efficient compared to other state-of-the-art clustering algorithms ($n = 20$ different seeds). Data are presented as mean values +/- 2SD.

To benchmark Forest Fire Clustering on a variety of single-cell sequencing technologies and data scales, we applied our method to analyze SHARE-seq (gene expression and chromatin accessibility) of mouse skin cells [2]. First, we preprocessed the gene expression count matrix with the "zheng17" recipe (using the top 10,000 genes) with the scanpy package in Python. With mouse skin cell marker genes provided by *Ma et. al. 2020*, we labeled jointly profiled cells using gene expression by using the "pg.infer_cell_types" function in the pegasus package in Python. Then, we performed the similar preprocessing on ATAC peak count matrix and used a variety of clustering methods to cluster the cells based on ATAC peak features.

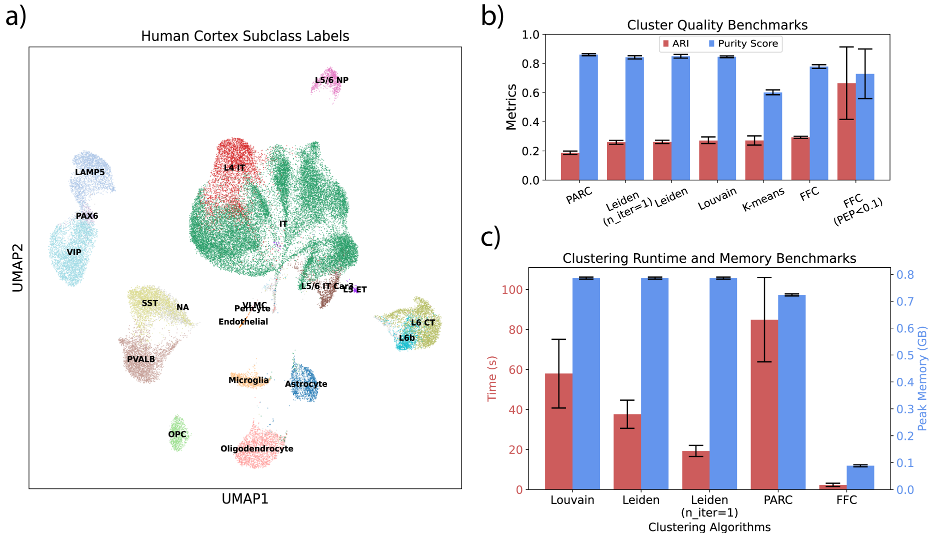

Supplementary Figure 12. Forest Fire Clustering Quality on Human Cortex: a) Gene expression with human cortex annotations from the Allen Brain Institute. Cell types were annotated using subclass labels constructed by subject matter experts. b) Forest Fire Clustering obtained higher quality clusters compared to existing methods, and Forest Fire internal validation improved the purity of the clusters ($n = 20$ different seeds). c) Runtime and memory usage benchmarks indicate that Forest Fire Clustering is faster and more efficient compared to other state-of-the-art clustering algorithms ($n = 20$ different seeds). Data are presented as mean values +/- 2SD.

Human cortex SMART-seq from the Allen Cell Type Database were also used to evaluate the performance of Forest Fire Clustering [3]. We first preprocessed the gene expression count matrix with the "zheng17" recipe (using the top 5,000 genes) with the scanpy package in Python. With subclass labels provided by *Hodge et. al. 2019*, we compared the accuracy and efficiency of Forest Fire Clustering with other clustering algorithms. Here, Forest Fire Clustering obtained similar high quality clusters in terms of ARI and purity score compared to other algorithms. Since many cortex cell types are well-defined and separable in manifold space, the clusters generated all have high purity scores. However, internal validation still improved the quality of Forest Fire Clusters compared to other clustering algorithms in terms of ARI.

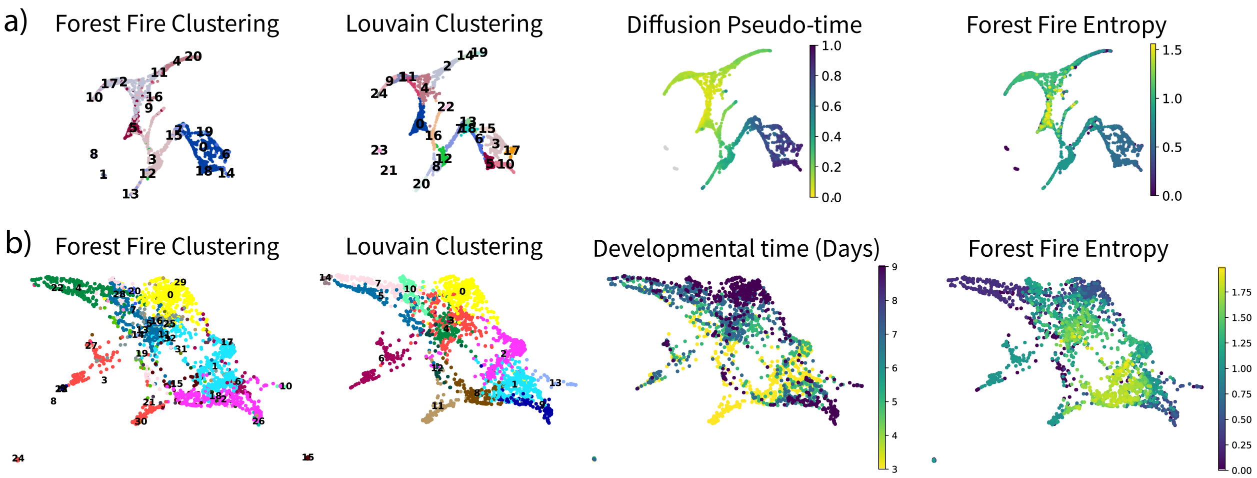

Supplementary Figure 13. Cell Path Diffusion Pseudo-time Analysis: PAGA topology maps were generated using Forest Fire clusters. a) For the *Paul et al.* dataset, Louvain clusters with mHSC signature gene expression (*Procr*) were selected as roots for diffusion pseudo-time. Without making assumptions on signature gene expression, Forest Fire entropy found key transition cells and highlighted similar developmental trajectories as diffusion pseudo-time. b) For the *Moon et al.* dataset, differentiation patterns of hESCs in real developmental time (3,5,7,9 days) were revealed using Forest Fire entropy without prior knowledge of early stem cell types.

For cell path analysis, *Paul et al.* data were downloaded using the Scanpy package, and *Moon et al.* were downloaded via Mendeley. Both datasets were preprocessed using the "zheng17" recipe and were clustered with Forest Fire Clustering (with adaptive kernel $K = 50, c = 75$) and Louvain (with $k = 50, resolution = 1$) on the top 50 principal components. In *Paul et al.*, Louvain clusters 0 and 16 were identified as stem cells by *Procr* mark expression for diffusion pseudo-time analysis. Forest Fire Monte Carlo was performed for 2000 rounds in both datasets, and point-wise entropies were calculated from posterior label distributions. To better visualize the developmental manifold, we used Forest Fire clusters to construct partition-based graph abstraction (PAGA) topology maps [4].

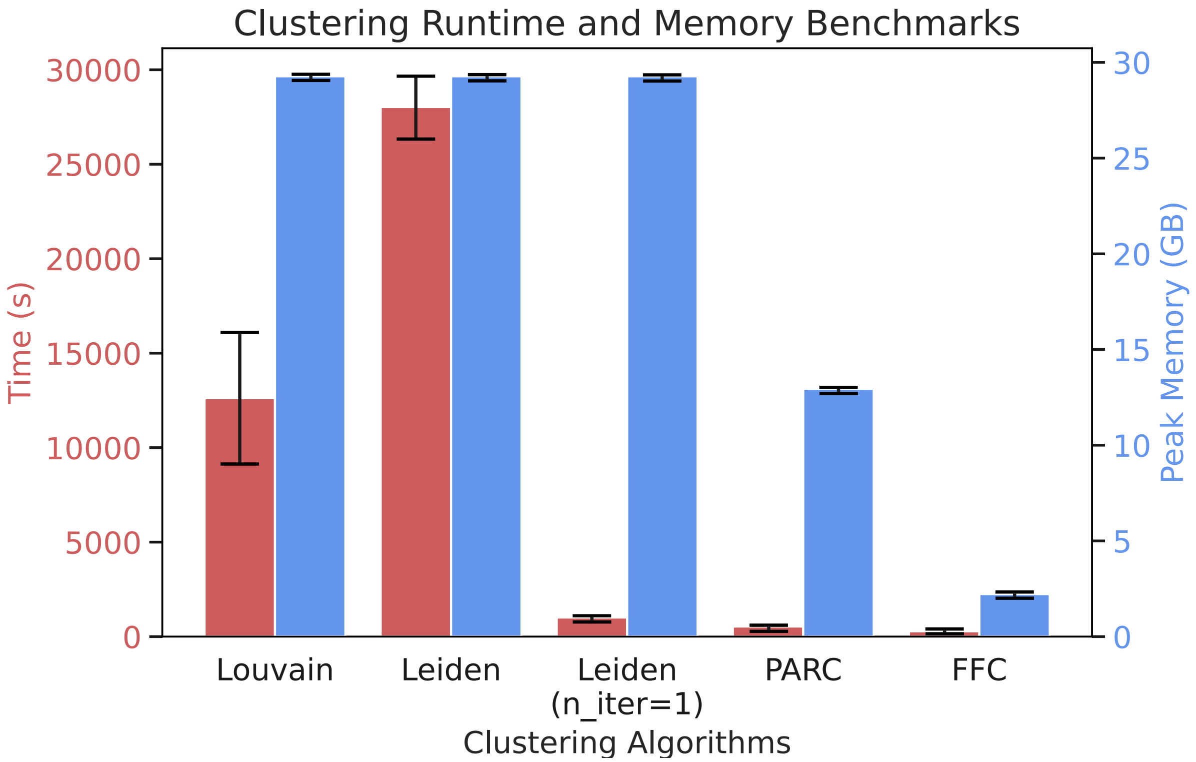

Supplementary Figure 14. 1.3 Million Mouse Brain Cell Runtime and Peak Memory Usage Benchmarks: Comparison on the runtime and peak memory usage of Forest Fire Clustering ($K = 100, c = 200$) with Louvain, Leiden, and PARC (resolution= 0.9, $K = 100$ using the first 30 principal components) on the $n = 1,300,000$ Mouse Brain Dataset from 10x Genomics. Data are presented as mean values +/- 2SD.

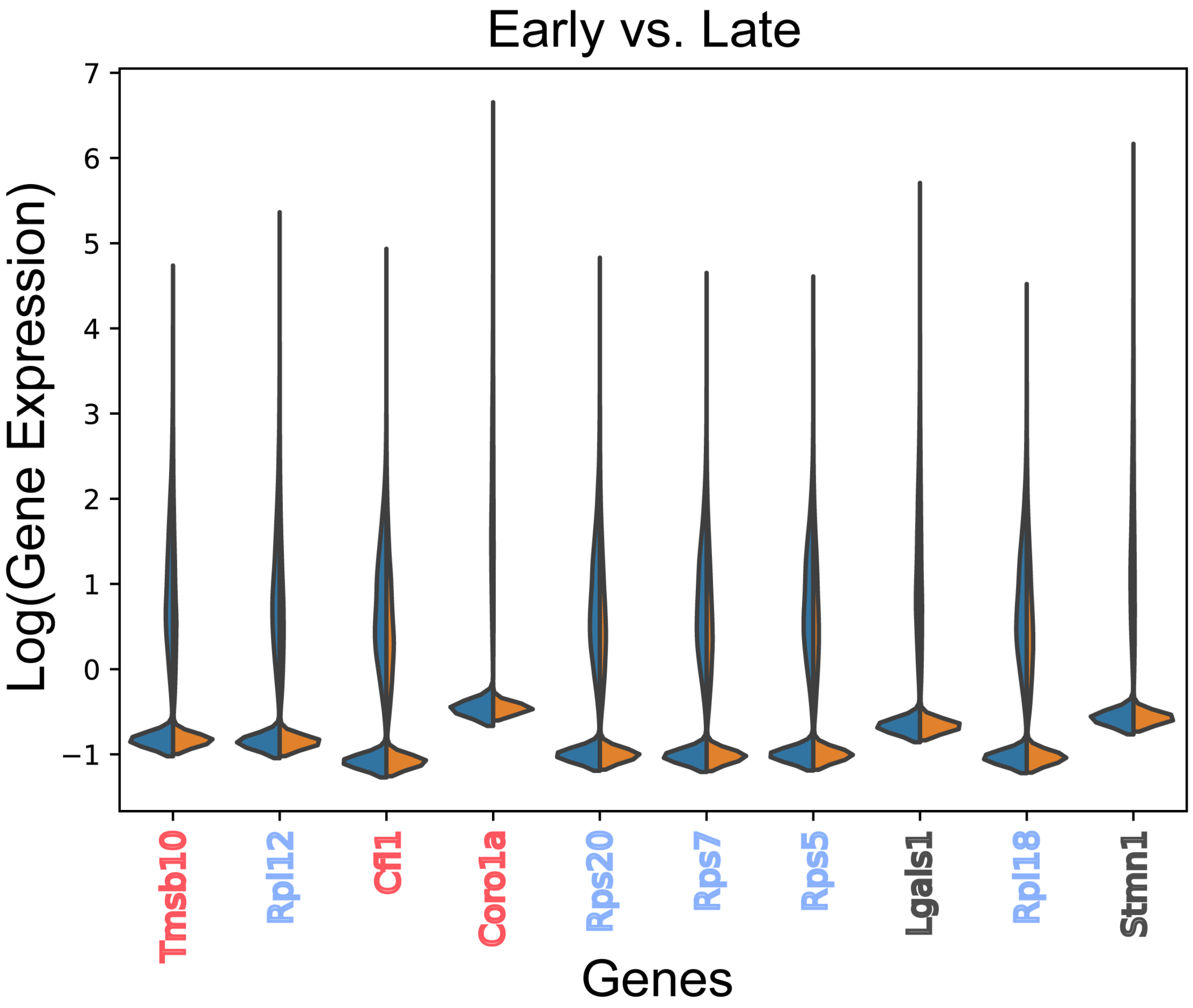

Supplementary Figure 15. Differentially Expressed Genes in Early vs Late MCA Cells: Top 10 differentially expressed genes in early vs. late MCA cells. Here, early and late cells are separated by using the median label entropy. Genes highlighted in red are related to actin binding, and genes highlighted in blue are related to rRNA binding. The top differentially expressed genes in early cells are all critical for organism development.

Gene	GO Molecular Function	Developmental Studies
Tmsb10	Actin Monomer Binding	Zhang, Xin, et al. "**Thymosin beta 10 is a key regulator of tumorigenesis and metastasis and a novel serum marker in breast cancer.**" Breast Cancer Research 19.1 (2017): 1-15.
Rpl12	Ribosome Subunit	Imami, Koshi, et al. "**Phosphorylation of the ribosomal protein RPL12/uL11 affects translation during mitosis.**" Molecular cell 72.1 (2018): 84-98.
Cfl1	Actin Binding	Zhu, Huiping, et al. "**Association between CFL1 gene polymorphisms and spina bifida risk in a California population.**" BMC medical genetics 8.1 (2007): 1-8.
Coro1a	Actin Binding, Cytoskeleton Binding	Punwani, Divya, et al. "**Coronin-1A: immune deficiency in humans and mice.**" Journal of clinical immunology 35.2 (2015): 100-107.
Rps20	Ribosome Subunit, RNA Binding	Nieminen, Taina T., et al. "**Germline mutation of RPS20, encoding a ribosomal protein, causes predisposition to hereditary nonpolyposis colorectal carcinoma without DNA mismatch repair deficiency.**" Gastroenterology 147.3 (2014): 595-598.
Rps7	RNA Binding	Watkins-Chow, Dawn E., et al. "**Mutation of the diamond-blackfan anemia gene Rps7 in mouse results in morphological and neuroanatomical phenotypes.**" PLoS genetics 9.1 (2013): e1003094.
Rps5	RNA Binding	Zhang, Xin, et al. "**Matrine attenuates pathological cardiac fibrosis via RPS5/p38 in mice.**" Acta Pharmacologica Sinica 42.4 (2021): 573-584.
Lgals1	Carbohydrate Binding	Li, Ji-Min, et al. "**Upregulation of LGALS1 is associated with oral cancer metastasis.**" Therapeutic advances in medical oncology 10 (2018): 1758835918794622.
Rpl18	Ribosome Subunit, RNA Binding	Chen, Cheng, et al. "**The nuclear gene rpl18 regulates erythroid maturation via JAK2-STAT3 signaling in zebrafish model of Diamond–Blackfan anemia.**" Cell death & disease 11.2 (2020): 1-11.
Stmn1	Tubulin Binding	He, Xiaoying, et al. "Elevated STMN1 promotes tumor growth and invasion in endometrial carcinoma." Tumor biology 37.7 (2016): 9951-9958.

Supplementary Figure 16. Function of Top Differentially Expressed Genes in Early MCA Cells: Gene name, gene ontology molecular function, and relevant studies of the top 10 differentially expressed genes (from Supplementary Figure 15) in early MCA cells

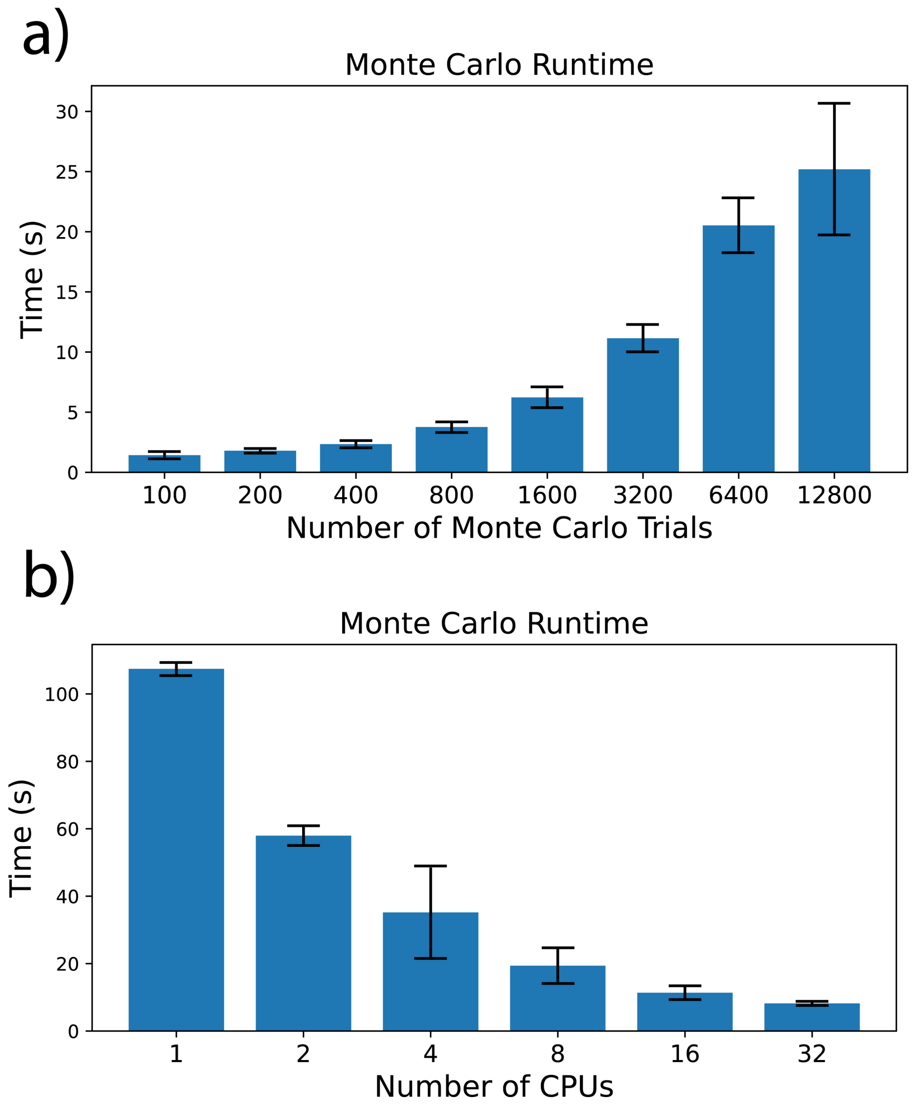

Supplementary Figure 17. Monte Carlo Runtime and Parallelization Analysis: a) The runtime of the Monte Carlo simulations increases linearly with the number of trials ($n = 20$ different seeds). b) The runtime of the Monte Carlo simulations decreases linearly with the number of CPUs. Since each simulation is embarrassingly parallel, we could parallelize the simulations with minimum synchronization overhead ($n = 20$ different seeds).

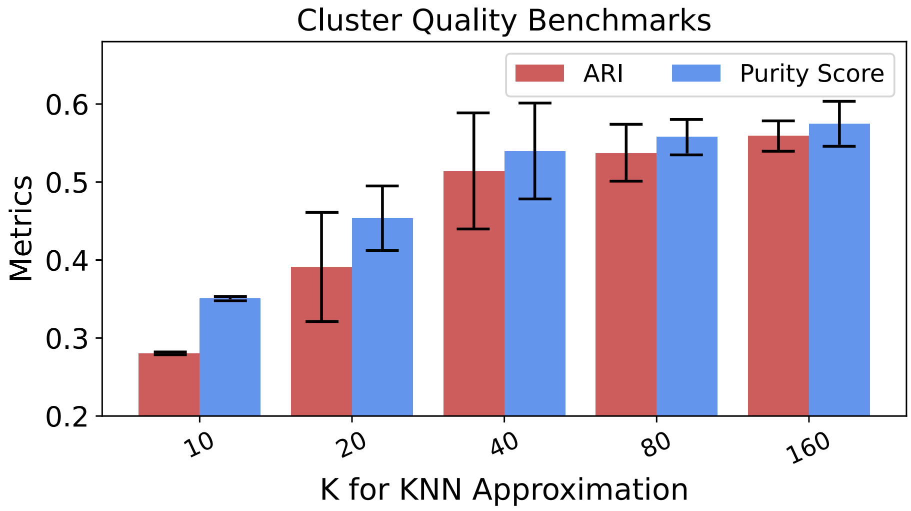

Supplementary Figure 18. Forest Fire Clustering Quality on PBMC for Different K in Approximating Neighborhood Graphs: We varied the K parameters when using a K-nearest Neighbor graph to approximate the affinity matrix in representing the data manifold ($n = 20$ different seeds). The result indicates the cluster quality increases as K increases in the beginning. However, after a certain threshold, increasing K only resulted in a marginal increase in clustering quality, indicating that K-nearest Neighbor graphs after a certain threshold can accurately represent the data manifold. Data are presented as mean values +/- 2SD.

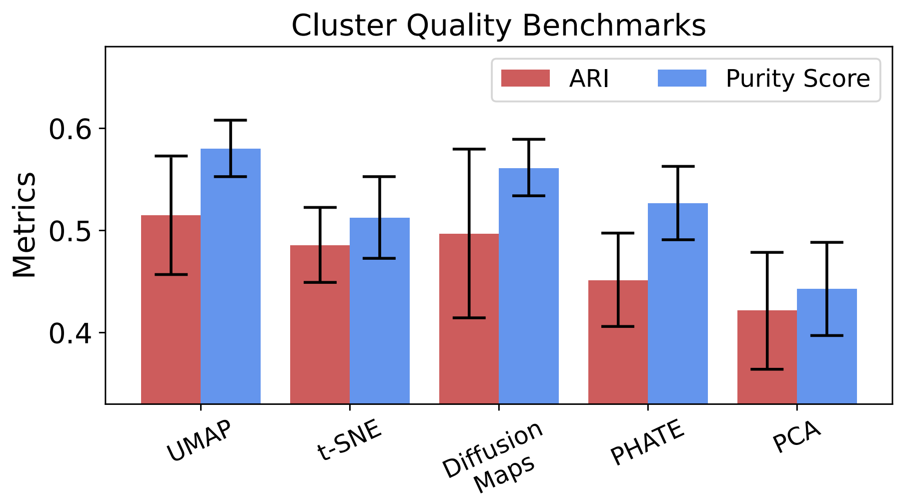

Supplementary Figure 19. Forest Fire Clustering Quality on PBMC for Various Dimensionality Reduction Methods: To reduce the dimensionality for Forest Fire Clustering, we used various types of low-dimensional (2D) embeddings for clustering ($n = 20$ different seeds). Here, the results indicate that UMAP obtained the best quality clusters compared to other methods. Data are presented as mean values +/- 2SD.

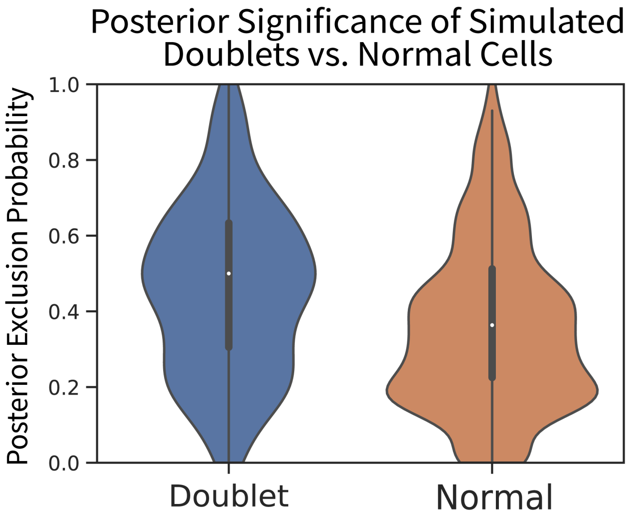

Supplementary Figure 20. Posterior Exclusion Probability for Doublet Detection and Quality Control: Artificial doublets were created by adding read-counts of each gene for cells from different clusters in the *Harris et al.* dataset ($n = 3,663$). Internal validation shows that doublet cells have higher posterior exclusion probabilities, indicating that point-wise posterior exclusion probabilities can be a useful metric for quality control. However, Forest Fire Clustering is a clustering algorithm, and doublet detection should be performed in the preprocessing step using doublet detection methods like Scrublet or DoubletFinder [5, 6]. Box plot visualizes data based on the minimum as lower whisker, first quartile or 25 percentile as lower box bound, median as center, third quartile or 75 percentile as higher box bound, and maximum as higher whisker.

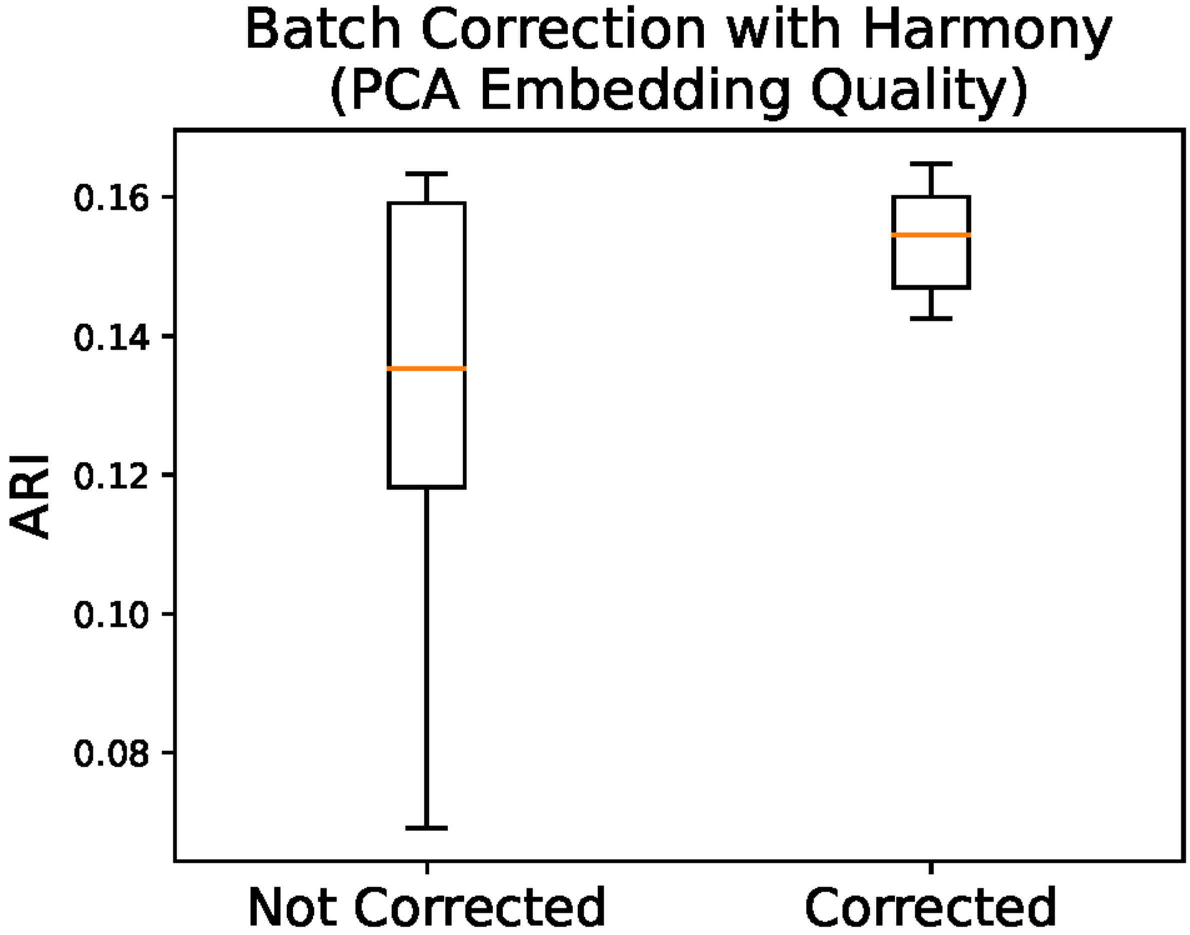

Supplementary Figure 21. Harmony Batch Correction on PBMC with Cell Surface Labels: Quantifying the effects of cell surface label quality on Harmony batch corrected PCA embeddings vs. uncorrected PCA embeddings [7] on Harris et al. ($n = 3,663$). Cell surface labels increased in ARI after batch correction with Harmony. Box plot visualizes data based on the minimum as lower whisker, first quartile or 25 percentile as lower box bound, median as center, third quartile or 75 percentile as higher box bound, and maximum as higher whisker.

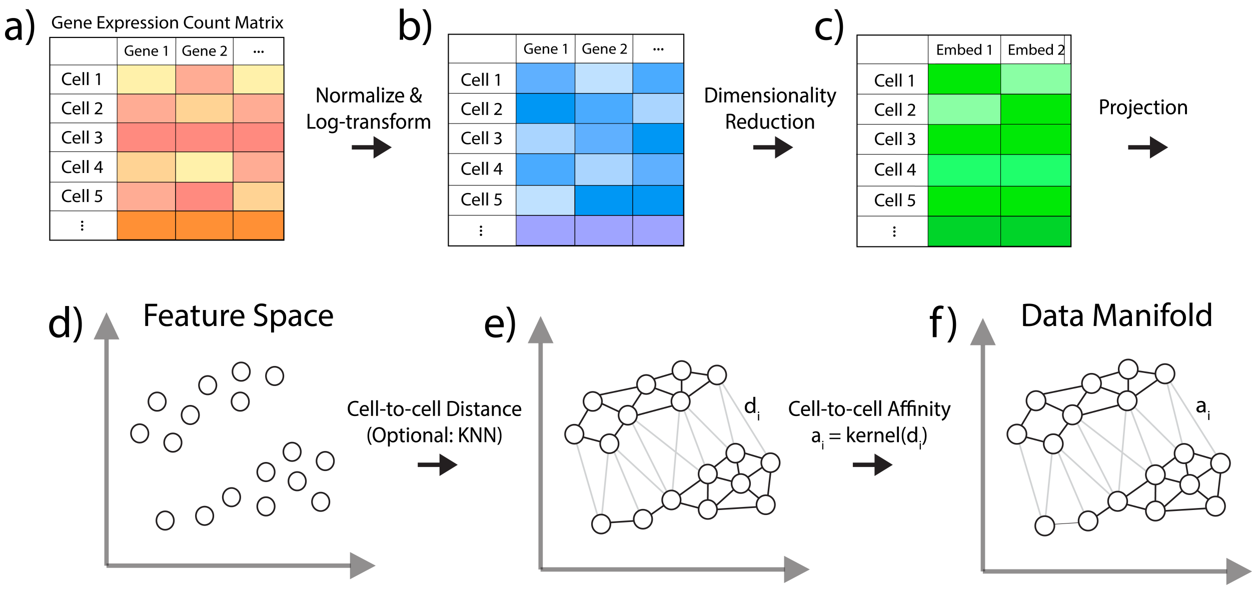

Supplementary Figure 22. Flowchart of the Preprocessing Steps: The preprocessing steps for single-cell analysis. a) Normalize and log-transform the gene expression count matrix. b) Select the top variable genes. c-d) Reduce the dimensionality of the data. e) Calculate the cell-to-cell distance. f) Kernel transform distances into affinities.

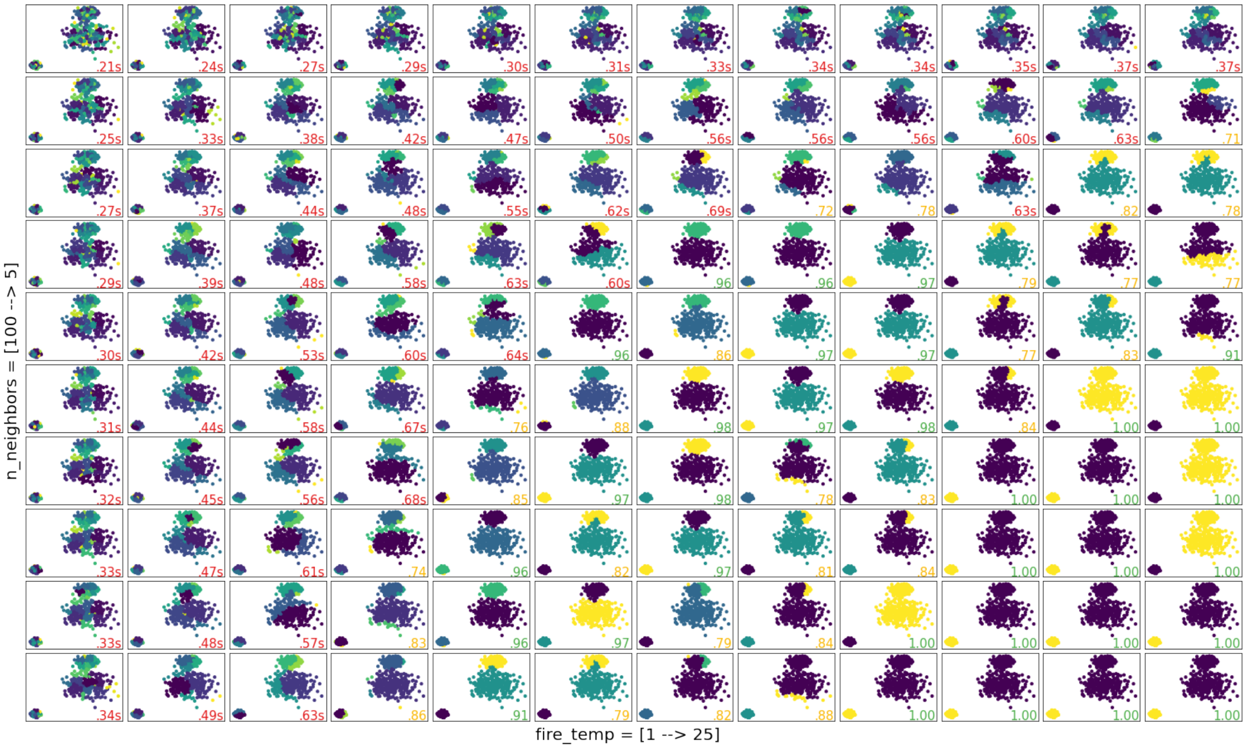

Supplementary Figure 23. Forest Fire Clustering Hyperparameter Configurations on Clustering: On a synthetic dataset, we used different combinations of the *n_neighbors* and *fire_temp* hyperparameters to investigate their effects on clustering. We also organized the code that generated this figure into an interactive notebook for users to test out different parameter configurations. With normalized and standardized features, we recommend default parameters $K = 50$ and $c = 50$, with fire temperature c ranging from 20 to 100, where increasing fire temperature decreases the cluster size.

Clustering Methods	Hyperparameters	Dimensionality Reduction Methods
K-means	K = [2, 4, 6, 8, 10]	PCA, PHATE, Diffusion Maps, t-SNE, UMAP
Louvain	resolution = [0.05, 0.1, 0.5, 1, 5]	
Leiden	resolution = [0.05, 0.1, 0.5, 1, 5]	
PARC	resolution = [0.05, 0.1, 0.5, 1, 5]	
Forest Fire Clustering	Fire temperature = [20, 40, 60, 80, 100]	

Supplementary Figure 24. Forest Fire Clustering Hyperparameter Configurations for Benchmarking: The combination of hyperparameter configurations and dimensionality reduction methods used for benchmarking.

1 References

1. Haghverdi, L., Lun, A. T. L., Morgan, M. D. & Marioni, J. C. Batch effects in single-cell rna-sequencing data are corrected by matching mutual nearest neighbors. *Nature Biotechnology* **36**, 421–427 (2018). URL `https://doi.org/10.1038/nbt.4091`.

2. Ma, S. *et al.* Chromatin potential identified by shared single-cell profiling of rna and chromatin. *Cell* **183**, 1103–1116 (2020).

3. Hodge, R. D. *et al.* Conserved cell types with divergent features in human versus mouse cortex. *Nature* **573**, 61–68 (2019).

4. Wolf, F. A. *et al.* Paga: graph abstraction reconciles clustering with trajectory inference through a topology preserving map of single cells. *Genome biology* **20**, 1–9 (2019).

5. Wolock, S. L., Lopez, R. & Klein, A. M. Scrublet: computational identification of cell doublets in single-cell transcriptomic data. *Cell systems* **8**, 281–291 (2019).

6. McGinnis, C. S., Murrow, L. M. & Gartner, Z. J. Doubletfinder: doublet detection in single-cell rna sequencing data using artificial nearest neighbors. *Cell systems* **8**, 329–337 (2019).

7. Korsunsky, I. *et al.* Fast, sensitive and accurate integration of single-cell data with harmony. *Nature methods* **16**, 1289–1296 (2019).
