# Supplementary material for: Forest Fire Clustering for single-cell sequencing combines iterative label propagation with parallelized Monte Carlo simulations
Source: Nat Commun. 2022 Jun 20;13:3538. doi: 10.1038/s41467-022-31107-8 (PMC9209427; doi:10.1038/s41467-022-31107-8)
Supplement: Supplementary file 2 — Reporting Summary [file 41467_2022_31107_MOESM2_ESM.pdf]

nature research

Corresponding author(s): Mark Gerstein

Last updated by author(s): May 18, 2022

Reporting Summary

Nature Research wishes to improve the reproducibility of the work that we publish. This form provides structure for consistency and transparency in reporting. For further information on Nature Research policies, see our Editorial Policies and the Editorial Policy Checklist.

Statistics

For all statistical analyses, confirm that the following items are present in the figure legend, table legend, main text, or Methods section.

n/a	Confirmed	
☐	☒	The exact sample size (*n*) for each experimental group/condition, given as a discrete number and unit of measurement
☐	☒	A statement on whether measurements were taken from distinct samples or whether the same sample was measured repeatedly
☐	☒	The statistical test(s) used AND whether they are one- or two-sided<br>*Only common tests should be described solely by name; describe more complex techniques in the Methods section.*
☒	☐	A description of all covariates tested
☐	☒	A description of any assumptions or corrections, such as tests of normality and adjustment for multiple comparisons
☐	☒	A full description of the statistical parameters including central tendency (e.g. means) or other basic estimates (e.g. regression coefficient) AND variation (e.g. standard deviation) or associated estimates of uncertainty (e.g. confidence intervals)
☐	☒	For null hypothesis testing, the test statistic (e.g. *F*, *t*, *r*) with confidence intervals, effect sizes, degrees of freedom and *P* value noted<br>*Give P values as exact values whenever suitable.*
☐	☒	For Bayesian analysis, information on the choice of priors and Markov chain Monte Carlo settings
☒	☐	For hierarchical and complex designs, identification of the appropriate level for tests and full reporting of outcomes
☐	☒	Estimates of effect sizes (e.g. Cohen's *d*, Pearson's *r*), indicating how they were calculated

Our web collection on statistics for biologists contains articles on many of the points above.

Software and code

Policy information about availability of computer code

Data collection	Synthetic data was generated using the scikit-learn (or sklearn) Python package (version 0.22.2) and Splatter (version 1.20) R package. Further, we collected data from published sources, with more details available in the "Data" section below.
Data analysis	We preprocessed the data with single-cell data processing libraries in Python 3.6 such as scanpy (version 1.8) and pegasus (version 1.2). Clustering was performed using algorithms implemented in scikit-learn (or sklearn) package (version 0.22.2), python-louvain (version 0.16), leidenalg (version 0.8.2), and our forest-fire-clustering library (version 0.0.25). Our Forest Fire Clustering algorithm was implemented with scanpy (version 1.8) for data preprocessing, numpy package (version 1.18.1) for linear algebra operations, the scipy package (version 1.4.1) for sparse matrix acceleration, and the numba package (version 0.53.1) for numerical acceleration at runtime. The source code for the Forest Fire Clustering library can be found on Github at https://github.com/gersteinlab/forest-fire-clustering. Further, clustering metrics for benchmarking were computed using functions implemented in scikit-learn (or Sklearn) package (version 0.22.2).

For manuscripts utilizing custom algorithms or software that are central to the research but not yet described in published literature, software must be made available to editors and reviewers. We strongly encourage code deposition in a community repository (e.g. GitHub). See the Nature Research guidelines for submitting code & software for further information.

Data

Policy information about availability of data

All manuscripts must include a data availability statement. This statement should provide the following information, where applicable:

- Accession codes, unique identifiers, or web links for publicly available datasets
- A list of figures that have associated raw data
- A description of any restrictions on data availability

Synthetic data were simulated using sklearn (in Python), and scRNA-seq data were simulated with Splatter (in R). Experimental scRNA-seq data were downloaded

from existing public repositories. CITE-seq and multiomic sequencing of PBMC datasets are available from https://www.10xgenomics.com/resources/datasets. The Mouse Cell Atlas dataset is also publicly available to download from http://bis.zju.edu.cn/MCA/.

Field-specific reporting

Please select the one below that is the best fit for your research. If you are not sure, read the appropriate sections before making your selection.

- [x] Life sciences
- [ ] Behavioural & social sciences
- [ ] Ecological, evolutionary & environmental sciences

For a reference copy of the document with all sections, see nature.com/documents/nr-reporting-summary-flat.pdf

Life sciences study design

All studies must disclose on these points even when the disclosure is negative.

Sample size	For single-cell RNA-sequencing experiments, the sample size is determined by the number of cells sequenced, where each cell is labeled by a unique molecular identifier. Using this metric as the sample size, PBMC CITE-seq and multiomics sequencing contains around 10,000 cells. The Mouse Cell Atlas contains around 370,000 cells.
Data exclusions	We used top 1k, 5k, or 10k highly variable genes in our analysis to emphasize the differences between cells in the single-cell RNA-seq datasets. Further, we only utilized cells with more than 10 read counts.
Replication	Each task was replicated for more than 20 times to obtain error bars for each statistic calculated in the manuscript.
Randomization	Since our experiment does not concern clinical trials or other biological experiments, randomization is not relevant to our study. Instead, we used benchmarking to compare against other computational methods.
Blinding	Since our experiment does not concern clinical trials or other biological experiments, blinding is not relevant to our study. Instead, we used benchmarking to compare against other computational methods.

Reporting for specific materials, systems and methods

We require information from authors about some types of materials, experimental systems and methods used in many studies. Here, indicate whether each material, system or method listed is relevant to your study. If you are not sure if a list item applies to your research, read the appropriate section before selecting a response.

Materials & experimental systems

n/a	Involved in the study
[x]	[ ] Antibodies
[x]	[ ] Eukaryotic cell lines
[x]	[ ] Palaeontology and archaeology
[x]	[ ] Animals and other organisms
[x]	[ ] Human research participants
[x]	[ ] Clinical data
[x]	[ ] Dual use research of concern

Methods

n/a	Involved in the study
[x]	[ ] ChIP-seq
[x]	[ ] Flow cytometry
[x]	[ ] MRI-based neuroimaging
